# Covid-19-related health literacy: a cross-sectional study in Israel: Michal Laron

**DOI:** 10.1093/eurpub/ckac131.094

**Published:** 2022-10-25

**Authors:** N Abdel-Rahman, M Laron, O Baron-Epel, T Artom, D Levin-Zamir

**Affiliations:** 1 Health Policy Team, Myers JDC Brookdale, Jerusalem, Israel; 2 School of Public Health, University of Haifa, Haifa, Israel; 3 Department of Health Education and Promotion, Clalit Health Services, Tel Aviv, Israel

## Abstract

**Background:**

The “infodemic” related to Covid-19 emphasized the importance of the public’s ability to access, understand, appraise and use information to make decisions about health. This study aimed to:

1. Assess the components of Covid-19 related health literacy (Co-HL)

2. Examine the associations of socio-demographic variables and health status with Co-HL

**Methods:**

This study was conducted as part of the European Health Literacy Population Survey 2019-2021 (HLS19). A cross-sectional survey of a representative sample of adults in Israel was conducted in December 2020 using phone interviews and an online survey (n = 1,315). Five items measuring Co-HL were added. Multivariable regression models were used to assess the associations between socio-demographic variables and health status with Co-HL.

**Results:**

Of participants, 63% reported concern about Covid-19. The mean general HL was lower among those who reported concern about Covid-19 compared to those who worried less (p = 0.002). The most difficult component of Co-HL was “judging the reliability of information regarding Covid-19” (36% expressed difficulty). Older participants, those with low self-reported social status, and low self-assessed health, were significantly (p < 0.05) more likely to express difficulty in judging the reliability of Covid-19 information. Interestingly, education level was not significantly associated with Co-HL.

**Conclusions:**

Our results suggest that, to best promote the use of information on Covid-19 prevention, older people, those with low social status and those with poor general health should be prioritized for improving critical health literacy.

**Key messages:**

• Co-HL is unequally distributed in the population, warranting tailored health promotion efforts.

• It is vital to improve the ability of the population to identify reliable information about covid-19.

